# Safe range of femoral neck system insertion and the risk of perforation

**DOI:** 10.1186/s13018-023-04205-6

**Published:** 2023-09-19

**Authors:** Mingxuan Han, Cong Li, Ning Han, Guixin Sun

**Affiliations:** Department of Traumatic Surgery, Shanghai East Hospital, School of Medicine, Tongji University, Shanghai, China

**Keywords:** Femoral neck fracture, Femoral neck section, Digital simulation, Safe zone

## Abstract

**Background:**

Internal fixation of the femoral neck carries a risk of perforation due to the presence of the isthmus of the femoral neck. At present, there are few studies on the safe and risk zones of the femoral neck system (FNS) implantation. This study aimed to recommend the safe range of injection of FNS in the lateral wall of the proximal femur, parallel to the axis of the femoral neck, during FNS treatment of femoral neck fracture (FNF).

**Methods:**

Femoral computed tomography (CT) data of 80 patients (male: 40; female: 40) who met the inclusion criteria were collected. Mimics 21.0 software was used to complete the modeling. 3-Matic 13.0 software was used to establish the axis of the femoral neck and its vertical plane, perform the cutting of the femoral neck, and project it on the vertical plane of the femoral neck axis. After matching a rectangle for each projection map, all sample sizes (80 cases) were standardized and superimposed to obtain gradient maps of the safe zone (SZ) and dangerous zone (RZ), thereby securing edge key points and safe FNS insertion range.

**Results:**

In the 80 samples, the mean diameter of the smallest femoral neck section was 33.87 ± 2.32 mm for men and 29.36 ± 1.92 mm for women. All 80 femoral necks had safe and risky areas. The SZ/S × 100% was 77.59 (± 2.22%), and the RS/S × 100% was 22.39% (± 2.22%). The risk area was composed of four parts: (1), (2), (3), and (4), respectively, corresponding to 3.45 ± 1.74%, 5.51 ± 2.63%, 6.22 ± 1.41%, and 7.22 ± 1.39%. Four marginal key points, perforation risk, and safe ranges (SR) of FNS were analyzed on the lateral wall of the femoral neck.

**Conclusions:**

The SR of FNS placement was recommended by digital simulation. In addition, Regions (3) and (4) posed a higher risk of penetrating the cortex. Using the gradient map of RZ for preoperative evaluation is recommended to avoid iatrogenic perforation.

## Background

**Fig. 1:**
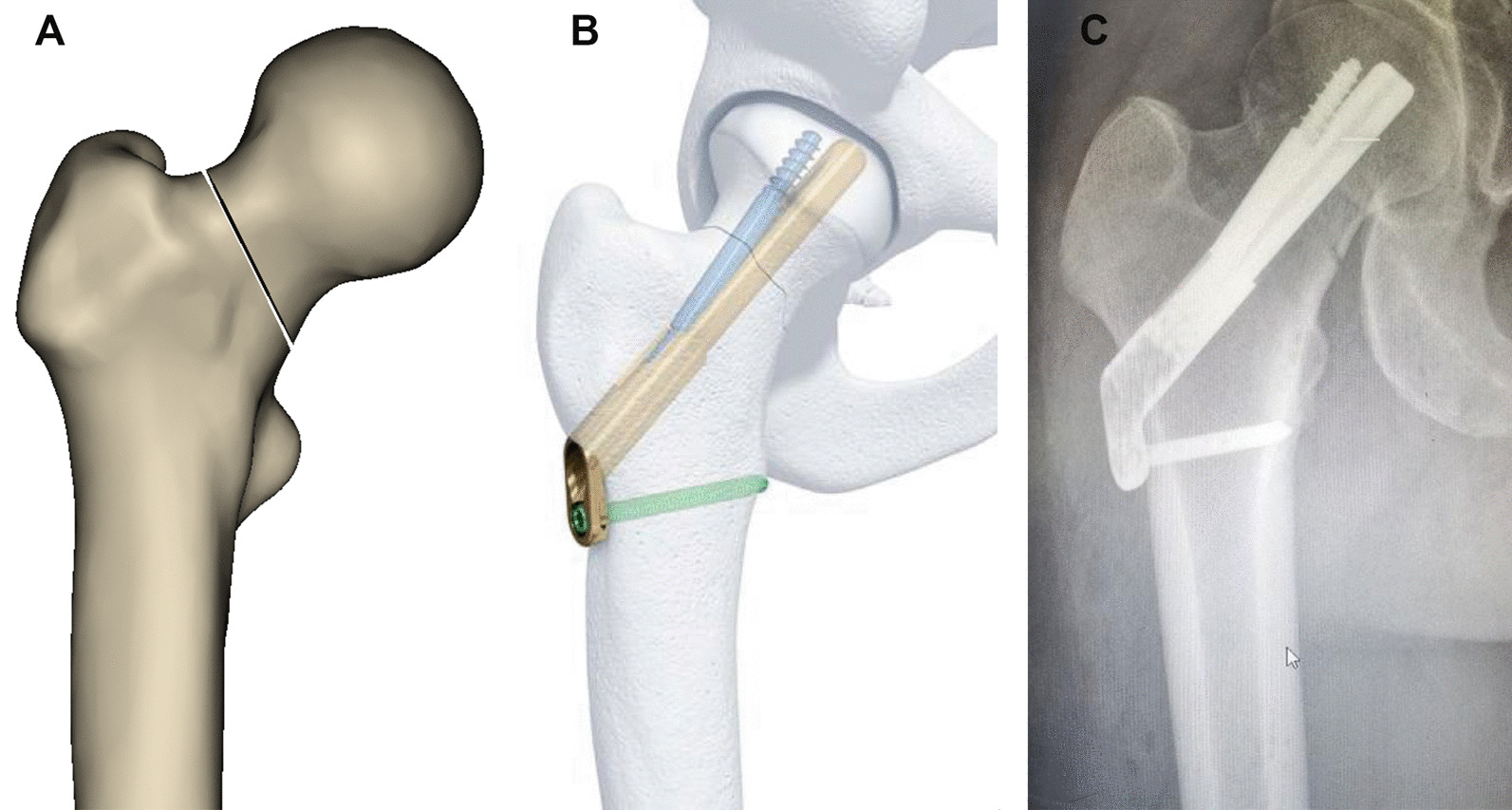
Femoral neck fracture model (**A**), internal fixation model of the new internal fixation femoral neck system (**B**), and X-ray visualization of FNS for femoral neck fracture (**C**)

The femoral neck is a crucial stress concentration site in the proximal femur and a frequently occurring location for fractures. Femoral neck fractures (FNFs) account for approximately 50% of hip fractures [1]. Regarding damage mechanism, FNFs in young patients are typically caused by high-energy injuries such as car accidents or falls from heights [2]. However, in the elderly population, FNFs are frequently attributed to low-energy injuries such as falls [3–5]. Treatment modalities vary based on patient age and fracture type. According to The American Academy of Orthopedic Surgeons Evidence-Based Guideline on Management of Hip Fractures in the Elderly [6]: Moderate evidence supports operative fixation for patients with stable (nondisplaced) FNFs. Strong evidence supports arthroplasty for patients with unstable (displaced) FNFs. For young patients with FNFs, the service life of the artificial hip joint is limited, and secondary revision surgery is both difficult and costly. Therefore, internal fixation should be prioritized as the primary treatment option to preserve the femoral head and maximize hip joint function recovery. Common methods for the internal fixation of FNFs include cannulated compression screws (CCS), dynamic hip screws (DHS), and femoral neck system (FNS) (Fig. 1). The selection of an appropriate internal fixation device is crucial to the success rate of surgery and the incidence of postoperative complications. However, controversy still exists regarding the optimal choice of internal fixation devices for treating FNFs. FNS is a novel technique for treating FNFs that offers reduced trauma, enhanced stability against rotation and shearing forces, and decreased risk of screw cutting, nail removal, and other complications [7]. Surgeons can achieve minimally invasive surgery while simultaneously reducing the size of implants used [8, 9]. Moreover, a superb biomechanical study has demonstrated that the stability of FNS is equivalent to that of DHS when an anti-rotation screw is used [10]. Compared with CCS, FNS has demonstrated a reduction in intraoperative fluoroscopies, radiation exposure for both medical staff and patients, and short-term complications such as femoral neck shortening and bone nonunion [11]. FNS for FNFs enhances postoperative hip functional recovery, reduces the rate of femoral neck shortening, and minimizes fluoroscopy exposure [12]. During surgery, adjusting the guide needle repeatedly to achieve a satisfactory internal fixation position may cause bone destruction. This is particularly problematic since the proximal femur is mostly cancellous bone, which makes patients with osteoporosis more vulnerable to bone mass loss and reduced bone strength, These factors can result in decreased internal fixation holding force, fracture end displacement, and, ultimately, internal fixation failure [13]. Such subtle changes may not be accurately analyzed from the x-ray data. In addition, the C-arm machine used for intraoperative fluoroscopy to assess the position of the guide needle provides only two-dimensional images, which mainly rely on the subjective assessment of the position by the surgeon, and some errors are inevitable. When the FNS is driven into the femoral neck along the guide pin, there is a risk of the guide pin not being properly positioned, and the isthmus of the femoral neck being present, which can lead to the FNS penetrating the femoral neck cortex. Different experimental techniques are used to improve the accuracy of the placement and reduce the incidence of perforation-related complications. These techniques include plain radiography, computed tomography (CT) scanning, and three-dimensional reconstruction. They are used to evaluate and measure the isthmus of the femoral neck and provide projections of different sections of the femoral neck along their axis to the lateral wall of the femur. This helps to determine the safe zone (SZ) for FNF placement. It also provides a safe range of entry points along the femoral neck axis, as safe entry points allow for safer screw placement and reduce the risk of perforation of the femoral neck cortex.

## Material and methods

This study was approved by the ethics committee of Shanghai Oriental Hospital. It was a retrospective investigation of medical imaging data. All patient data were anonymized, and the study was performed with informed consent from all patients.

### Sample data

The inclusion criteria were as follows: (1) patients aged 18 years and above and (2) hip or femur CT examination in the imaging center of our hospital (Shanghai East Hospital, School of Medicine, Tongji University.) during 2022–2023. The exclusion criteria included incomplete imaging, posttraumatic proximal femoral deformities or congenital abnormalities, and patients with a history of previous trauma or proximal femoral surgery. This study was conducted with consent of volunteers and approved by the Shanghai East Hospital Ethics Committee.

## Research method

### Three-dimensional reconstruction of the femur model

Mimics 21.0 medical imaging software was used to convert the imported CT scan data into a three-dimensional reconstruction model of the femur.

### Murphy's method to identify the axis of the femoral neck

**Fig. 2:**
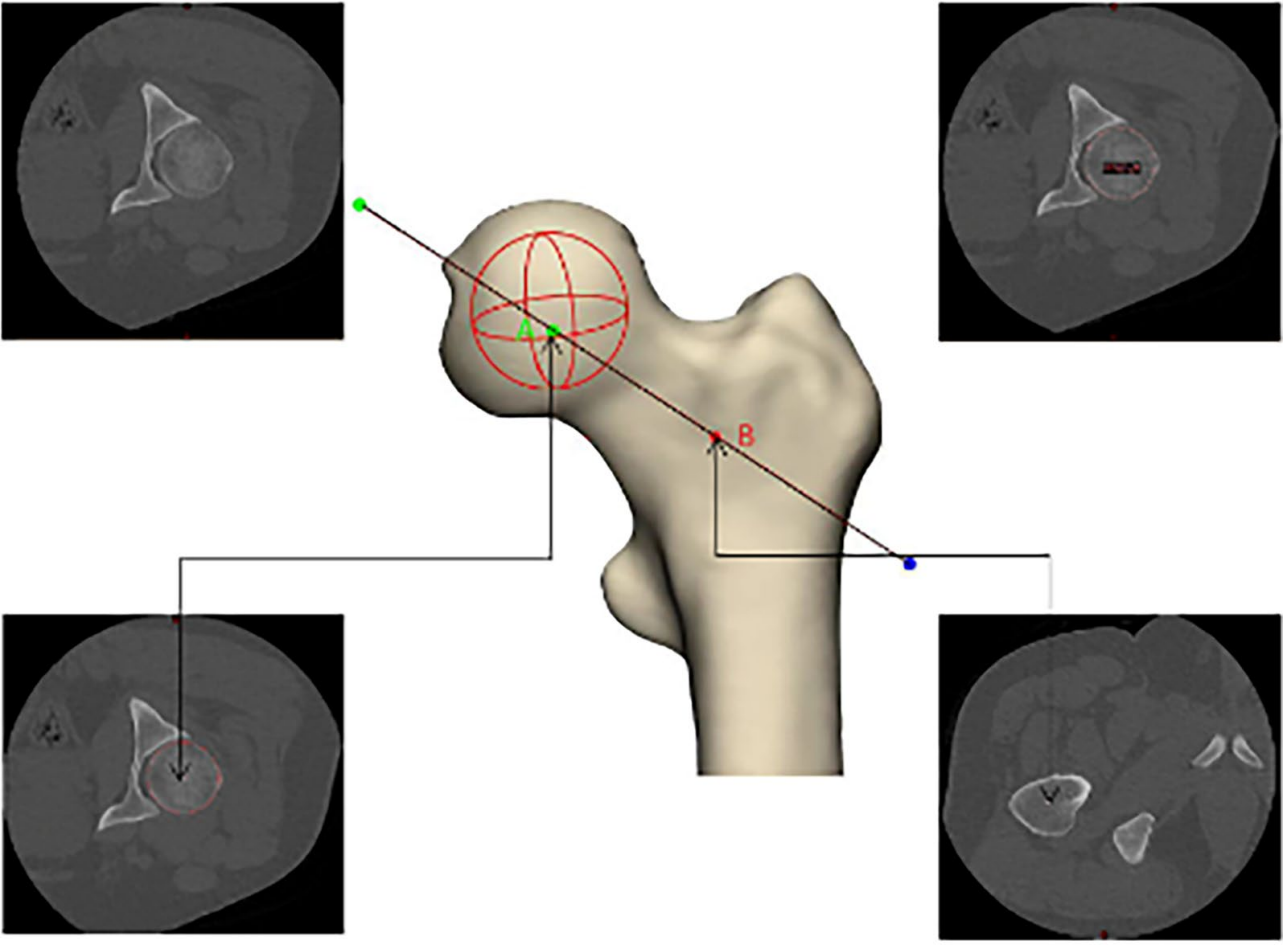
Murphy’s method identifies the axis of the femoral neck. The top left is a random CT plan of the femoral head; The figure on the upper right shows the diameter of the femoral head on the CT plane. The figure at the lower left is the maximum diameter of the femoral head in all CT planes (the center of the circle in CT plane is marked as **A**, that is, the center of the femoral head in the fitting sphere). The lower right figure is a CT plan of the base of the neck of the femur (the upper margin of the lesser trochanter, roughly triangular, and the center of the triangle is **B**, the center of the base of the neck of the femur)

The data of two CT cross-sections were determined, namely the maximum cross-section of the femoral head and the base cross-section of the femoral neck. The center of the maximum section of the femoral head and the center of the base section of the femoral neck were selected and marked. Connecting these two points was the axis of the neck of the femur determined by Murphy's method (Fig. 2).

### Obtaining a cross-section of the neck of the femur

**Fig. 3:**
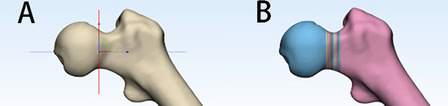
Before (**A**) and after (**B**) femoral neck cutting

A plane perpendicular to the femoral neck axis was created at the femoral head–neck junction, called the moving plane. The moving surface was translated along the axis of the femoral neck using the moving plane function. The Cut function was used layer by layer until the moving surface was tangent to the greater trochanter for every 1 mm of translation (Fig. 3). The diameter of the femoral neck section was recorded for each sample.

### Creation of a projection map along the femoral neck axis

**Fig. 4:**
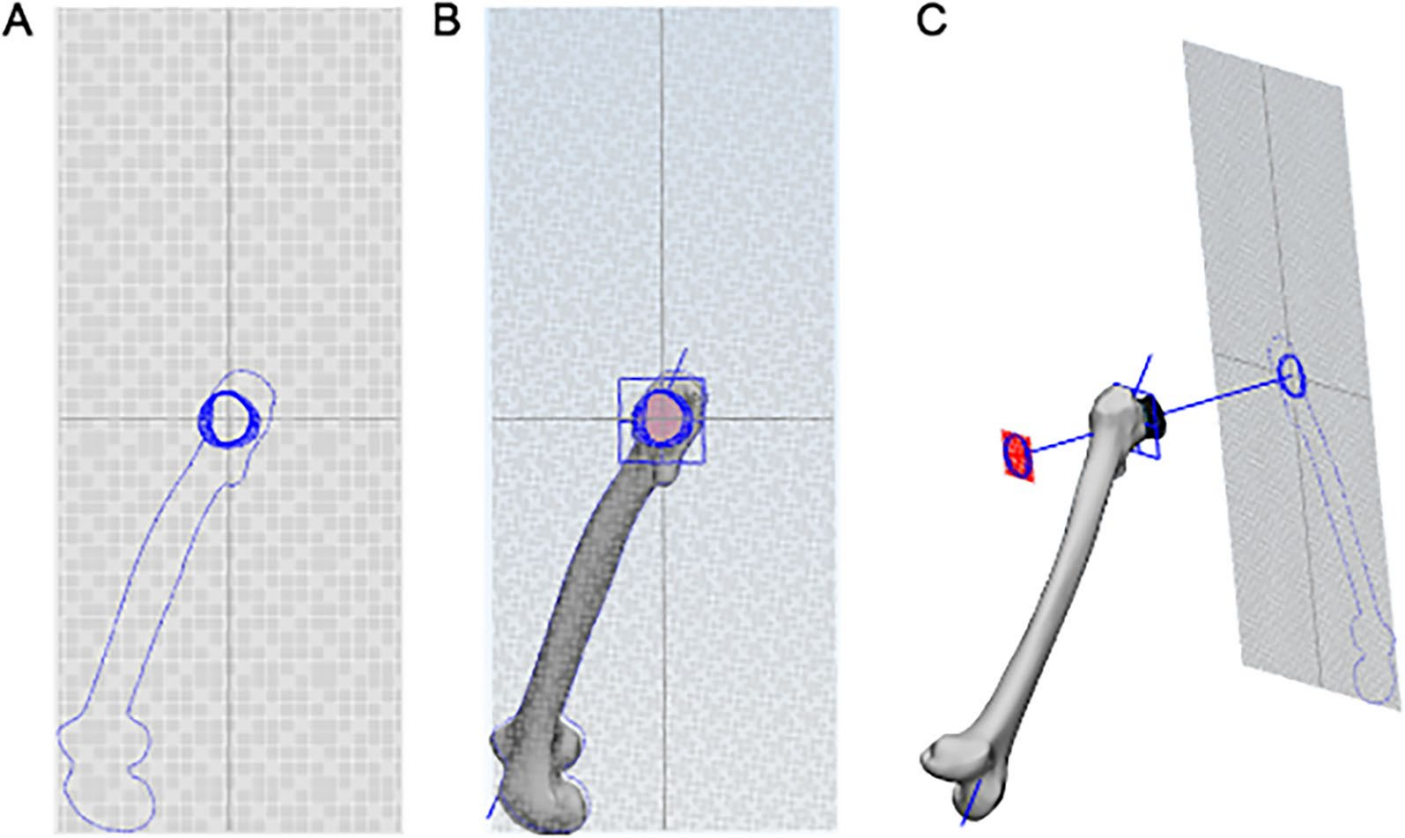
Projection map along the axis of the femoral neck, with the femur hidden (**A**), the femur shown (**B**), and the position relationship between the femur and the projection plane (**C**)

After measuring and recording the diameters of all the fault sections, they were projected onto the vertical plane of the femoral neck axis to obtain a projection set of all the sections. At the same time, a projection set on the lateral femur was obtained (Fig. 4).

### Creation of coordinates along the femoral neck axis

**Fig. 5:**
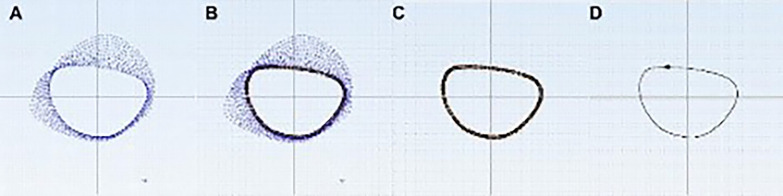
The safe zone of femoral neck was obtained by the projection map of femoral neck section. The projection of femoral neck section (**A**) Outlines the closed curve along the innermost side (**B**), the Delete Geometry command is applied to obtain the separate curve (**C**), and the Refine Spline command and Convert to Context Curves command are used to obtain the true smooth curve (**D**)

Finally, the projection of the femoral neck section onto the lateral wall of the femur was obtained along the femoral neck axis. The innermost curve was outlined as shown in Fig. 5.

### Axial diagram analysis of the femoral neck

SZ was defined as the position of the screw in the femoral neck (anteroposterior views of the femoral neck and axial views) on the x-ray film and was actually located in the cortex. RZ was defined as only conforming to the former and actually penetrating the cortex. Perforation risk was defined as the ratio of the RZ/S area.

**Fig. 6:**
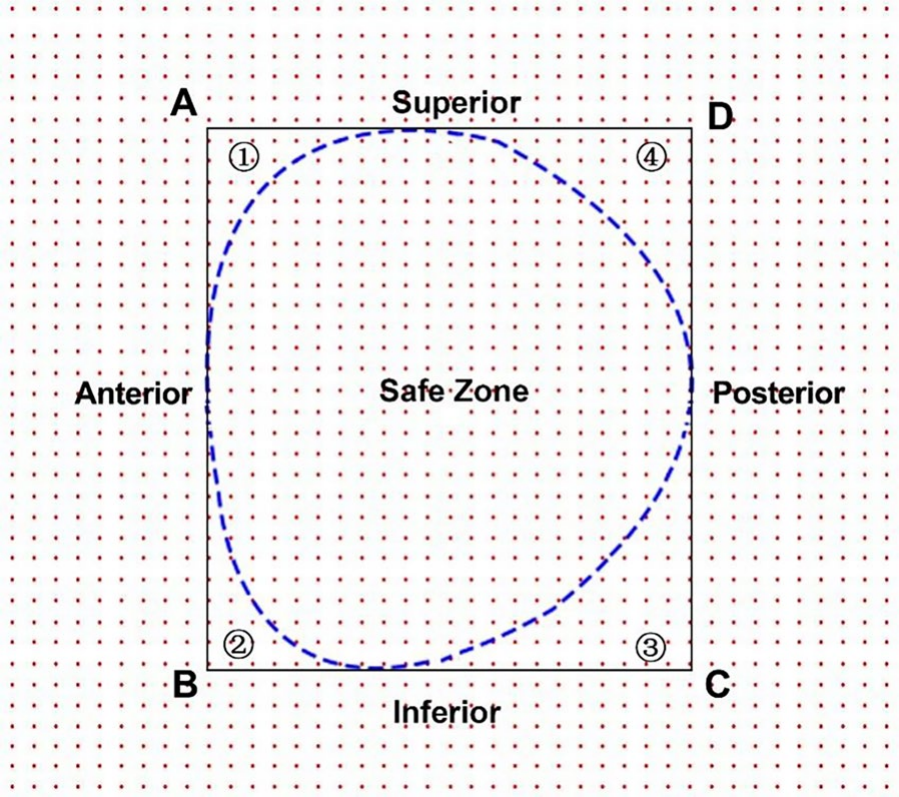
A projective graph along the femoral neck axis. The rectangle was regarded as S; SZ is the neck projection (real SZs); ①②③④ are RZs of penetration. RZ = 1 + 2 + 3 + 4; SZ = S-RZ. In the axial diagram, a rectangle determined by 4 dots (**A**: boundary anterosuperior,** B**: boundary anteroinferior,**C**: boundary posteroinferior，**D**: boundary posterosuperior), was referred to as S

**Fig. 7:**
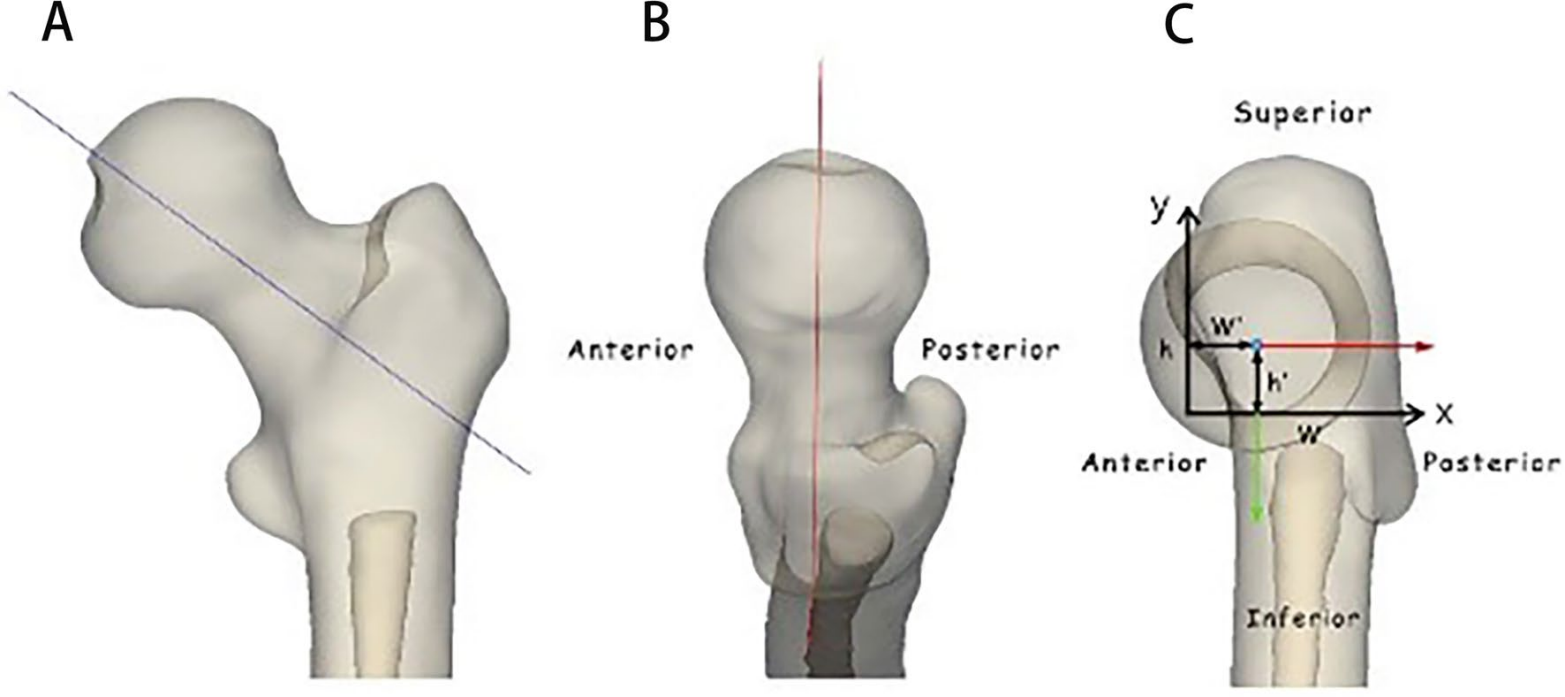
Simulated radiographs. AP view (**A**). Lateral view (**B**). The axial view (**C**) shows the relationship of the guide wire position and the AP, lateral and axis graphs. h’ is the distance from the guide wire to the inferior curve; h is the distance from the superior curve to the inferior curve; w’ is the distance from the guide wire to the anterior curve; w is the distance from the posterior curve to the anterior curve. *y* = *h*’/*h*, indicates screw position in the AP view. *x* = *w*’/*w*, indicates screw position in the lateral view

As mentioned earlier, a rectangle determined by four dots (ABCD) was referred to as S in the axial diagram (Fig. 6). The neck projective area was referred to as SZ. The remaining area of the rectangle was the RZ, which included four parts: (1), (2), (3), and (4) (Fig. 6). The following data were calculated and recorded: width and height of S, RZ/S × 100%, (1)/S × 100%, (2)/S × 100%, (3)/S × 100%, and (4)/S × 100%. Each original axial diagram was reshaped (zoomed) to match a calculated average shape. Reshaped axis graphs were generated with the same calculated average shape, allowing for the mapping and visualization of the RZs of all femoral necks in a single averaged axial graph. After all of the images were compiled into one image, a gradient of RZs was visualized, and a coordinate system was created [14, 15] (Fig. 7). The *x*-value on the horizontal axis indicated the bolt position on the lateral view. The *y*-value on the vertical axis indicated the bolt position on the AP view.

## Result

**Fig. 8:**
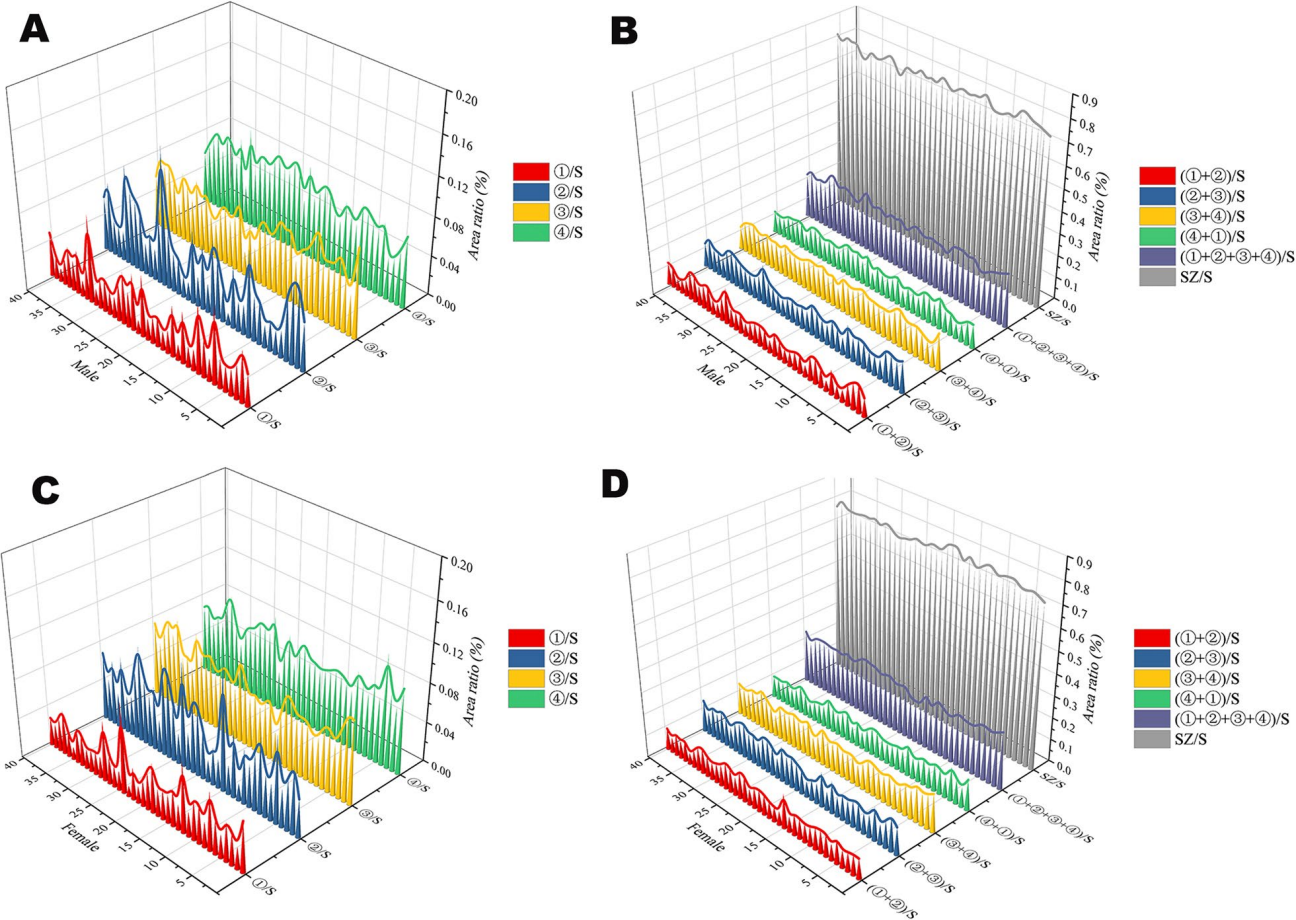
Plots of the percentage of each risk zone and safe zone for males and females are shown in **A**–**D**

In the 80 samples, the mean diameter of the smallest femoral neck section was 33.87 mm for males; For women, 29.36 mm; The distance from the location of the smallest section of the neck of the femur to the center of the neck of the femur: the mean distance for males is 20.87 mm, 19.17 mm for women. The average NSA of male was 128.72° in 80 samples. For women, it was 128.62°. There are safe and risky areas in all 80 femoral necks. SZ/S × 100% is 77.59(± 2.22%), the RS/S × 100% is 22.39% (± 2.22%). Risk area is composed of four parts: ①, ②, ③, ④ respectively corresponding to 3.45 ± 1.74%, 5.51 ± 2.63%, 6.22 ± 1.41%, 7.22 ± 1.39% (Fig. 8).

### Gradient of risk zones and key edge points

**Fig. 9:**
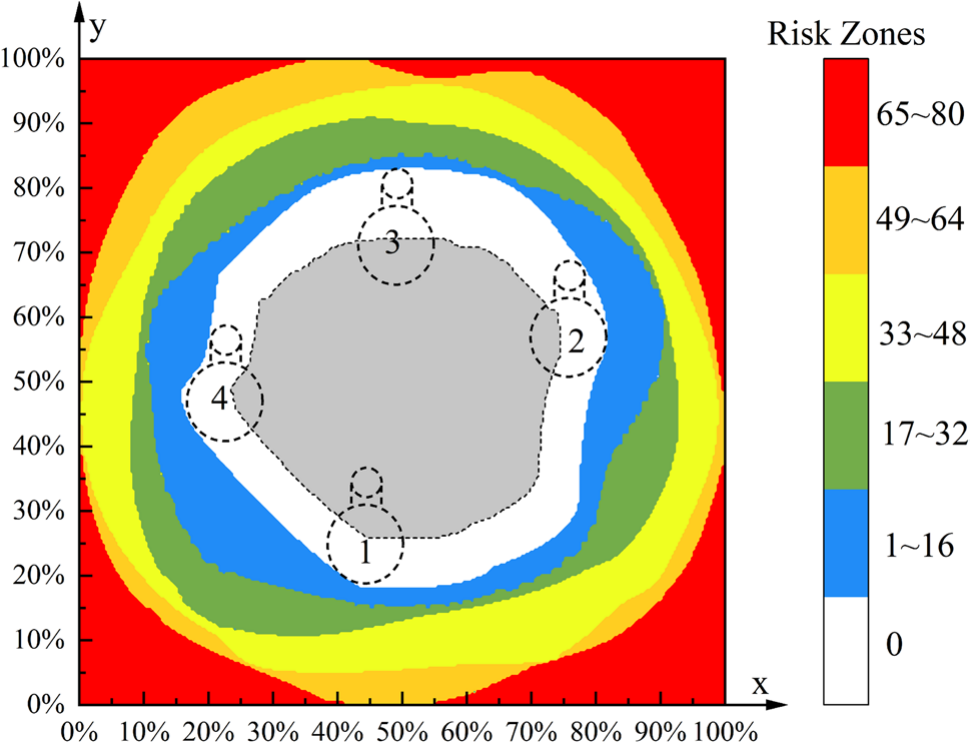
Gradient of risk zones. This figure reflects not only the positional relationship between the bolt and the antirotation screw, but also the correlation between their positions and the risk of femoral neck perforation. The distance between the axis of the femoral neck and the intersection point of the femoral head and the lateral wall of the femur was 92.29 ± 7.72 mm. According to FNA guidelines (92.29–5 = 87.29 mm), the length of the bolt (dashed circle with a diameter of 10.0 mm) was 85 mm, and the anti-rotation screw with the same length of the bolt (dashed circle with a diameter of 6.4 mm) was selected. In addition, the four marginal key points of SR indicate that the 10 mm diameter bolt and 6.4 mm antirotation screw are safe to contact with the femoral neck cortex. Key point 1, *X* = 45%, *Y* = 26%; key point 2, *X* = 75%, *Y* = 57%; key point 3, *X* = 50%, *Y* = 72%; key point 4, *X* = 24%, *Y* = 49%

By adjusting and processing each original projection image, after reshaping, the projection is compiled into an image, generating a visual gradient and coordinate system for RZ. Identify the safe and cortex-touching zones (SCTZs) in the neck of the femur. The maximum safe trajectory of the center of the bolt and anti-rotation screw was established without penetrating the femoral neck cortex. The bolt and anti-rotation screw of the FNS are safe at the femoral neck and can contact the cortex. Four key marginal points were then marked using the neck axis of the femur as reference points (Fig. 9).

## Discussion

Although some studies suggest that the incidence of hip fractures may be stabilized or decreasing [16–18], there is still a significant concern about the increasing number of hip fractures due to the aging of the global population and the increase in life expectancy. The number of hip fractures is expected to reach 6.3 million by 2050 [19]. Hip fractures occur predominantly in elderly people, with the reported incidence in the United States of 414/100,000 and 957/100,000 in men and women, respectively. Postoperative complications occur in up to 49% of patients, and the first-year mortality is as high as 36% [20, 21]. FNFs account for approximately 50% of hip fractures [22]. Thus far, various treatment strategies have been used to treat FNF, including cannulated screw fixation, DHS, intramedullary nails, locking plates, and hip replacement. We rarely consider hip replacement in young patients because the life of the prosthesis is usually shorter than the life of the patient. If this surgery is performed, the patients are at a high risk of secondary surgery in the future, which undoubtedly increases the patient’s pain and financial burden. In addition, many serious complications can occur after hip arthroplasty, such as periprosthetic infection, dislocation, loosening of the prosthesis, and periprosthetic fracture [23, 24]. The most commonly used internal fixation strategies in young patients with FNFs are undoubtedly preserved hip surgeries, such as cannulated screw fixation and dynamic hip screw fixation.

The unique advantages of FNS have been clinically recognized and applied. This study focused on improving the accuracy of FNS placement and reducing the incidence of complications related to cortical perforation.

The angle formed by the intersection of the axis of the femur and the axis of the neck of the femur in the coronal plane is femoral neck shaft angle (FNSA). This angle is about 150° in infancy and between 125° and 135° in adulthood. The average is 127° for women and 132° for men. The presence of FNSA can make the femoral axis more inclined to the lateral pelvis to accommodate a larger range of hip motion. When the FNSA is less than 120°, the varus hip can increase the shear stress of the femoral neck. However, when the FNSA is greater than 140°, femoral neck eversion can increase the compressive stress. Studies suggest that when the neck-shaft angle increases by one standard deviation, the risk of proximal femoral fracture increases 2.45 times in men and 3.48 times in women. Therefore, the neck-shaft angle is extremely important when choosing internal fixation surgery. Postoperative angle recovery leads to a change in the direction of stress. It may lead to a stress fracture or prosthesis loosening after the surgery. It is important to measure FNSA correctly both preoperatively and during postoperative follow-up. Current methods for measuring the femoral neck stem angle include direct measurements from anatomical specimens, x-ray measurements, and CT 3D reconstruction measurements. In clinical practice, x-ray and CT stereoscopic reconstructions are commonly used to measure femoral neck trunk angles. Still, some scholars [25–27] concluded that the x-ray measured angle was too large. They argued that directly applying the x-ray-measured angle to the femoral neck backbone was highly likely to produce a significant bias. In this study, CT 3D reconstruction was used to measure the NSA. While the method overcomes the limitations of x-ray measurements and provides more accurate results, it is complex and comes with a higher economic cost. Hence, a simple and accurate measurement method needs to be developed.

Many factors affect the healing of the femoral neck. In any case, the anatomical reduction and the restoration of the blood supply of the femoral neck are the key factors for the healing of FNF. The results of the successful reduction and internal fixation of FNFs are as follows. The ultimate treatment goals are not only the healing of the fracture end of the femoral neck but also the absence of femoral head necrosis. Anatomical reduction requires good liner-positional alignment after reduction. Functional reduction is not allowed for FNFs,

which differs from femoral shaft fractures. The purpose of anatomical reduction is to allow the fracture to heal as soon as possible. Anatomical reduction is achieved when only the fracture line of the femoral neck is seen on the x-ray. Internal fixation does not pursue strong internal fixation, but pursues reliable internal fixation, that is, the stability of the broken end after internal fixation. After ensuring proper axial pressure, all other stresses that can cause instability of the broken end and head must be eliminated. In terms of biomechanics, FNS has certain advantages. The locking anti-rotation screw of FNS and the main screw are locked together, providing better anti-rotation effects. Moreover, the sliding compression function can be realized by sliding between the main screw and the lateral plate, promoting fracture healing.

Fracture reduction is a variable controlled by the surgeon. After sufficient induction of anesthesia, closed reduction is attempted. If closed reduction is unsuccessful, the surgeon must be prepared to perform open reduction because the quality of reduction is key to help avoid avascular necrosis [28, 29]. After successful reduction, precise internal fixation cannot be ignored. According to the FNS operating manual, the fracture is reduced by gentle traction/flexion, adduction/abduction, and internal rotation (greater than 15° so that the femoral neck is parallel to the operating table) after the patient lies supine on the operating table. A new guide wire is placed along the upper/anterior portion of the femoral neck to prevent unintended rotation of the femoral head. A 130° angular guide is used to insert a second, new guide needle as the central guide needle. The guide needle is placed below the apex of the femoral head under orthoscopy and continued into the subchondral bone. Laterally, the guide wire should be centered on the neck of the femur and the femoral head. The off-axis fixation of the femoral neck increases the strain area and total displacement of the bone, which, in turn, increases the risk of fixation failure. Therefore, the central placement of FNS may be a better surgical target in treating FNFs [30].

**Fig. 10:**
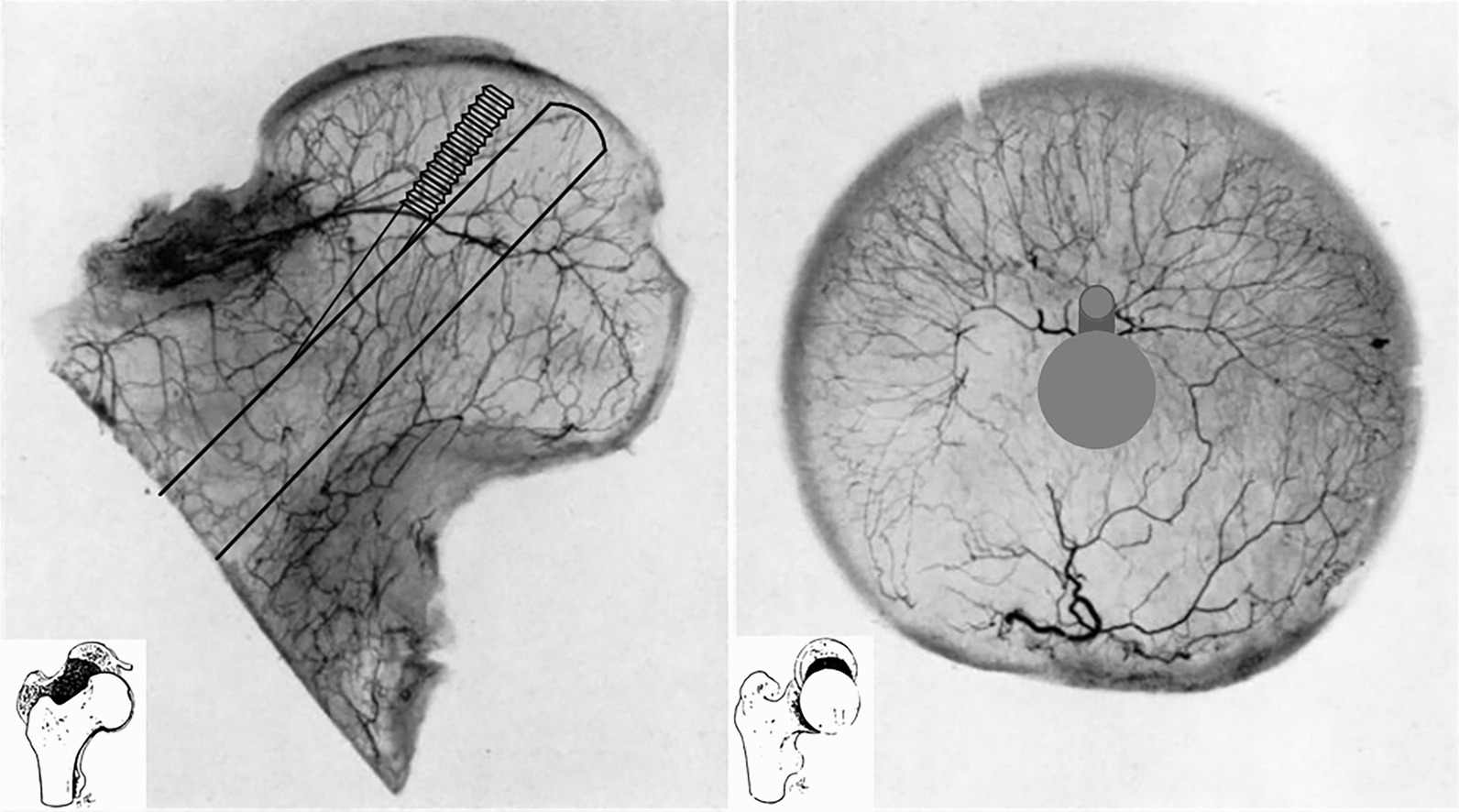
shows the pattern of internal artery and implant position in the coronal (left) and sagittal (right) planes of the femoral head after implantation of the FNS device

The bolt of FNS should be placed on the central axis of the femoral head and neck (Fig. 10). It requires the surgeon to accurately locate the screw during the surgery to ensure accurate placement. Whether FNS can be implanted in other positions below the central axis of the femoral head and neck is still unclear. Few studies have been conducted on different placement positions of FNS. It is difficult to ensure accurate placement during the surgery due to the harsh placement of the screw and the 10-mm diameter of the bolt of FNS, leading to the penetration of the femoral neck cortex and the destruction of the blood vessels. Among the four risk areas in this study, the risk proportion of quadrant (4) was the highest (7.22 ± 1.39%), implying that the most likely area of iatrogenic perforation of the femoral neck was located in quadrant (4). This might damage the blood supply to the femoral head and be closely related to the anatomy of the blood vessels of the femoral head and neck. A screw penetrating the posterior upper quadrant cortex of the femoral neck might impair the blood supply to the femoral head. Previous studies showed that the lateral epiphyseal artery stem was the most important blood supply vessel in the head. The inferior terminal stem of the synovium was located in the posterior upper quadrant of the femoral neck. A majority of vascular pores (77%) were also located in this quadrant [31]. Penetration of the femoral neck cortex disrupted these vital branches, interfering with revascularization and even leading to avascular necrosis of the femoral head. In 1953, Trueta [32] reported that the most important blood supply to the femoral head was the superior retinacular artery issued by the medial circumflex femoral artery. Its main artery rose to the lateral artery and entered the center of the femoral head between cartilage and bone marrow. It supplied at least two thirds of the volume of blood to the femoral head. It was close to the bone cortex, with high vascular tension and low mobility, and it was easy to injure this blood vessel when the femoral neck was fractured. The blood vessels that reached and were distributed in the femoral head were all small vessels after several branches. Despite anastomosis between them, they still maintained relatively independent blood supply areas.

In practice, absolutely precise positioning is difficult to achieve, as well as the correct position of small deviation does not significantly increase the risk of fixed failure, if appear the correct position of small deviation, we need to weigh the repeated the introduction to further injury of the surrounding tissue and repeated the perspective of the risk of radiation [33].

Zhao et al. [34] demonstrated that the central fixation directly facing the central region of the epiphyseal artery network avoided damage to the main stems of these epiphyseal arteries. The epiphyseal arterial stem near the peripheral cortex had a larger diameter than the network located in the central region (Fig. 10). Hence, the anastomosis frequency was less, and injury was prone to occur. The anastomosis was more and the compensatory ability was higher in the central region. The epiphyseal artery network and the inferior supporting artery system are important structures to maintain the blood supply of the femoral head after FNF. Enhanced protection of these critical structures during surgery, such as drilling and placement of internal implants close to the central region of the femoral head, may help reduce the impact of an iatrogenic injury on the intraosseous vascular system.

We must be familiar with the following definitions to minimize the effect of iatrogenic injury on blood vessels and cortical bone. The isthmus of the femoral neck was defined as the narrowest cross-section of the femoral neck (narrow FN) [35] perpendicular to the long axis of the femoral neck. This section was the only way for the implant to pass through, and it was also the most dangerous section for implant placement. However, in this study, we used the “cut-projection” method to obtain “narrow FN.” The author believes that the aforementioned method is more reasonable than direct measurement because the neck of the femur is not a smooth, flat, and regular stereogeometric figure. This “plane” obtained in this method is not the projection of a single cut fault, but rather the collection of all cut faults of the femoral neck at its closest points to the axis. These points exist in three-dimensional space, and their projections onto the vertical plane of the femoral neck axis converge into a two-dimensional plane.

In practice, the insertion point of the implant is located in the lateral cortical area of the proximal femur. Unnecessary iatrogenic injury can be effectively avoided only by establishing an SZ at the beginning of needle insertion. As shown in the axial view in Fig. 8C, the “narrow FN” coordinate system was established in the lateral axial view of the proximal femur after cancellations of the anteversion angle. All the “narrow FN” were analyzed and processed using Photoshop image processing software, and all the “narrow FNs” were “standardized” and then superimposed so as to obtain the “narrow FN in the final version” (Fig. 9, white area). According to the position relationship, the anti-rotation screw was located above the bolt, with the bolt and the anti-rotation screw as a whole. The maximum safe trajectory of the center of the bolt and the anti-rotation screw was established without penetrating the femoral neck cortex. Then, the femoral neck axis was used as the reference point to mark the four key edge points.

### Limitations

First, the sample size was relatively small. With only 40 males and 40 females, a larger analysis sample could be conducted to reduce potential sampling error. Second, whether the femoral anteversion angle has an effect on the direction of FNS insertion and the safety zone has not been fully described in this article. More in-depth studies are needed. Third, the choice of the implantation length of bolt and anti-rotation screw is obtained according to the average measurement of the sample size, which is inevitable to have measurement error, and in the actual operation process, the length of the two can be inconsistent. Finally, the bolt of FNS is required to be placed on the central axis of the femoral head and neck, which is undoubtedly harsh. It is not clear whether FNS is allowed to be placed at other positions below the central axis of the femoral head and neck. There are few biomechanical studies on different placement positions of FNS. To explore the ideal placement of FNS, evaluate the fault-tolerance ability of FNS, and provide reference for the effective application of FNS in the treatment of femoral neck fractures.

## Conclusion

In this paper, we came up with an average piercing risk of 22.39% (± 2.22%). In general, the probability of perforation increases from the center to the edge of the axial graph. This means that care should be taken to avoid cortical perforation when both intraoperative radiographs show the screw near the cortex, especially if the screw is located in the ③ or ④ quadrants. An accurate understanding of the RZ of the femoral neck will help the surgeon avoid iatrogenic cortical perforation, vascular injury, and cortical breakage. In summary, relying solely on AP and lateral radiography to predict implant placement during surgery still carries a risk of cortical perforation. The experimental results presented in this paper suggest that the risk of perforation is about 22%. In particular, the risk is greatest in the ④ region. In addition, implant placement is still related to biomechanical strength and vascular injury, which is also a component that surgeons need to consider in full. To reduce the risk of perforation and iatrogenic injury, a gradient map of the RZ is recommended for preoperative evaluation.

## Data Availability

The datasets used and/or analysed during the current study are available from the corresponding author on reasonable request.
